# Role of extracellular vesicles in the pathogenesis of mosquito-borne flaviviruses that impact public health

**DOI:** 10.1186/s12929-024-01096-5

**Published:** 2025-01-04

**Authors:** Pedro Pablo Martínez-Rojas, Verónica Monroy-Martínez, Blanca H. Ruiz-Ordaz

**Affiliations:** Departamento de Biología Molecular y Biotecnología, Instituto de Investigaciones Biomédicas, Universidad Nacional Autónoma de México (UNAM), 04510 Mexico City, Mexico

**Keywords:** Arbovirus, *Flavivirus*, Mosquito-borne flaviviruses, *Flavivirus* pathogenesis, Extracellular vesicles, Exosomes, Microparticles, Intercellular communication

## Abstract

Mosquito-borne flaviviruses represent a public health challenge due to the high-rate endemic infections, severe clinical outcomes, and the potential risk of emerging global outbreaks. Flavivirus disease pathogenesis converges on cellular factors from vectors and hosts, and their interactions are still unclear. Exosomes and microparticles are extracellular vesicles released from cells that mediate the intercellular communication necessary for maintaining homeostasis; however, they have been shown to be involved in disease establishment and progression. This review focuses on the roles of extracellular vesicles in the pathogenesis of mosquito-borne flavivirus diseases: how they contribute to viral cycle completion, cell-to-cell transmission, and cellular responses such as inflammation, immune suppression, and evasion, as well as their potential use as biomarkers or therapeutics (antiviral or vaccines). We highlight the current findings concerning the functionality of extracellular vesicles in different models of dengue virus, Zika virus, yellow fever virus, Japanese encephalitis virus, and West Nile virus infections and diseases. The available evidence suggests that extracellular vesicles mediate diverse functions between hosts, constituting novel effectors for understanding the pathogenic mechanisms of flaviviral diseases.

## Background

Flaviviruses are positive-sense RNA viruses that have emerged as pathogens and are transmitted by *Aedes*, *Hemagogus*, or *Culex* spp. mosquitoes; nonvector transmission methods involving sexual contact, from mother to child (in-utero or intrapartum), transfusion of blood components, organ transplantation, or body fluid exposure have been described [1, 2]. Infection cases present different clinical ranges from asymptomatic, mild self-limiting, or severe diseases characterized by hemorrhage, neuroinvasion, multiorgan compromise, or death [3, 4].

Dengue virus (DENV), Zika virus (ZIKV), yellow fever virus (YFV), Japanese encephalitis virus (JEV), and West Nile virus (WNV) represent the main mosquito-borne flaviviruses due to their continuing risk of infection in tropical and subtropical region inhabitants, constituting more than 50% of the world’s population. The rapid and efficient expansion of flaviviruses has occurred because of the high virulence of circulating strains, the presence of susceptible populations, and the wide distribution of vectors [5, 6]. As a result of the geographic distribution overlap, there is a possibility that the vectors can transmit more than one flavivirus at a time; thus, coinfection may directly impact the clinical outcome [7]. The viral cycle in mosquito vectors begins when the female acquires viruses during blood feeding. Viruses undergo replication and cross specialized epithelial barriers until they reach the salivary glands, where they are released into the saliva and transmitted to a new host during subsequent feeding; therefore, humans are inadvertent hosts [8, 9].

Flaviviruses exhibit different human cellular tropisms, which explains why these diseases present diverse clinical syndromes, such as flu-like illness, acute neurological disorders, hemorrhagic fever, and metabolic wasting [9]. Molecular and cellular events in vector–virus–human host interactions are critical for disease outcomes. Infected cells lead to stress signaling, which induces cell differentiation or activation with the release of extracellular vesicles (EVs) [10]. EVs [exosomes (small EVs) and microparticles (medium/large EVs)] are nanoparticles from cellular membranes that mediate intercellular communication by the transfer of proteins, nucleic acids, or lipids. In viral infections, EVs also transfer viral antigens, genomes, or complete virions, favoring transmission by receptor-independent mechanisms and triggering the inflammatory response or the suppression/evasion of the immune response. EVs may be potential biomarkers for disease progression or could be applied as nanotherapeutic tools [11–13].

This review focuses on the roles of EVs in DENV, ZIKV, YFV, JEV, and WNV infections. We highlight the evidence that demonstrates how EVs, through different study models, are involved as alternative mechanisms that may define mosquito-borne flavivirus pathogenicity.

## Epidemiology of mosquito-borne flaviviruses

According to the World Health Organization (WHO), the global incidence of DENV infections has markedly increased in the last two decades. From 2000 to 2019, the WHO documented a tenfold surge in cases worldwide. In 2023, over five million cases and more than 5000 dengue-related deaths were reported in over 80 countries in Africa, America, Southeast Asia, the Western Pacific, and Eastern Mediterranean regions [14].

As of 2022, more than 870,000 autochthonous ZIKV cases have been reported, and 89 countries have reported ZIKV transmission across the Americas, Africa, Southeast Asia, and the Western Pacific [15, 16].

YFV remains in a sylvatic cycle in tropical forests, although infection is prevented by a single dose of a safe vaccine [17]. In 2023, the WHO reported that 34 countries in Africa and 13 countries in Central and South America are endemic for YFV [18].

JEV is endemic to 24 countries in Southeast Asia and the western Pacific. There are an estimated 68,000 cases of JEV infection each year worldwide, and the case fatality rate can reach as high as 30% [19], whereas WNV is present in Africa, Europe, Asia (Middle East and West), and North America [20]. According to the Centers for Disease Control and Prevention (CDC) in the U.S., from 1999 to 2022, 56,575 human disease cases, 25,777 hospitalizations, and 2776 deaths were reported [21].

The increased incidence of flaviviruses and their introduction in new countries represent a serious health risk, in addition to population growth, industrialization, and vector adaptability due to environmental disturbances, such as climate change, promoting the conditions for probable future epidemics.

## Basic virology of flaviviruses: structure and replicative cycle features

The genus *Flavivirus* groups enveloped viruses of approximately 50 nm in diameter, surrounded by a lipid envelope, and encoded by a single-stranded, positive-sense RNA genome nearly 11 kilobases in length [22]. The viral genomic RNA encodes a single long open reading frame (ORF) flanked by the 5′ and 3′ untranslated regions (UTRs) involved in viral genome translation, replication, and packaging. The 5′ UTR (containing the type 1 cap m^7^GpppAmN) functions as the promoter for the initiation of RNA synthesis, and the 3′ UTR (which lacks a polyadenylated tail) is the precursor of the subgenomic flaviviral RNA (sfRNA) associated with pathogenicity, inhibition of antiviral responses, increased viral transmission, and increased replicative fitness [2, 23, 24].

The ORF is translated into a polyprotein, from which ten proteins are cleaved: three structural (capsid (C), membrane (M), and envelope (E)) proteins and seven nonstructural (NS) proteins. The C protein is involved in viral RNA packaging and nucleocapsid (NC) core formation; the M protein (a small proteolytic fragment of the precursor protein (prM) produced during viral maturation) functions as a chaperone for E protein folding and assembly; and the E protein mediates binding and fusion during viral entry. NS proteins (1, 2A, 2B, 3, 4A, 4B, and 5) are necessary for genome replication, viral assembly, and host immune response dysregulation [25, 26].

Flaviviruses exhibit broad cellular tropism, entering host cells through receptor-mediated endocytosis. It is known that they use a wide variety of receptors [22]. Viral endocytosis is followed by the fusion of the virion envelope with cellular membranes due to the low pH of the endosome. Endosome acidity triggers irreversible trimerization of the E protein, NC release into the cytoplasm, and C protein/RNA dissociation. The viral RNA is subsequently translated into a single polyprotein, which is cleaved into structural and NS proteins at the endoplasmic reticulum (ER) membrane. For viral RNA replication, a negative-sense strand is synthesized, serving as a template for new positive-strand genomes. The assembly process consists of the association of the C protein with genomic RNA, followed by budding into ER membranes that contain the E-prM protein complex, constituting immature virions. Virion maturation occurs in the trans-Golgi network, where proteolytic processing and acid-induced E-prM rearrangement promote prM cleavage. Additional steps, such as glycan modification, result in the formation of mature infective particles. Flaviviruses are released from host cells by exocytosis [2, 22–26].

The viral replication cycle requires viral proteins with the involvement of host molecules such as RNA-binding proteins (RBPs), which are ubiquitous in the cytoplasm and determine virus‒cell interactions [23–25]. Diosa-Toro et al. hypothesized that flavivirus RNA recognition relies on specific host RBPs that promote its incorporation at the EV cargo as an alternative mode for viral spread [24]. In this sense, the viral replication cycle and EV biogenesis may converge on common cellular mechanisms; this fact implies a determining role of EVs in flavivirus pathogenesis as an advantage of their ability to mediate cell-to-cell communication [27].

## Extracellular vesicles (EVs)

EVs are heterogeneous populations of particles released by cells, delimited by a lipid bilayer, and cannot replicate on their own [28]. In humans, EVs are present in all body fluids [29] and enclose and carry nucleic acids, proteins, and lipids that can modify behavior or trigger phenotypic changes in acceptor cells. The biological functions of EVs include removing (waste, harmful, or useless) intracellular components, sharing nutrients, and serving as cell-to-cell mediators; thus, they function in homeostasis and pathological processes [30–32].

The International Society for Extracellular Vesicles (ISEV) proposed the Minimal Information for Studies of EV (MISEV), which characterizes the physical properties of EVs, such as size, density, molecular composition, or cell origin. Exosomes are small EVs (sEVs) < 200 nm in diameter, and ectosomes (microparticles or microvesicles) can be medium/large EVs (m/lEVs) > 200 nm in diameter. Owing to their biogenesis, exosomes are released by exocytosis (through the endosomal pathway) of intraluminal vesicles (ILVs) from multivesicular bodies (MVBs), and ectosomes are released from the plasma membrane by budding [32, 33].

### EV biogenesis and uptake mechanisms

EV (exosome or ectosome) biogenesis occurs through different molecular machineries, such as the endosomal sorting complex required for transport (ESCRT), the tetraspanin signaling pathway, RBPs, sphingomyelinase-generating cone-shaped lipids (in the central nervous system, CNS), phospholipid relocalization toward the membrane leaflet, or actin cytoskeleton depolymerization [34].

Microparticles (MPs) are formed from the budding of the cell membrane due to the disruption of phospholipid asymmetry. Under physiological conditions, phosphatidylserine (PS) and phosphatidylethanolamine (PE) are located in the inner leaflet, whereas phosphatidylcholine and sphingomyelin are located on the outer membrane [35]. Phospholipid asymmetry is regulated by Ca^2+^-dependent enzymes such as flippases, floppases, scramblases, and translocases. The Ca^2+^ released from the ER enters the plasma membrane, activating enzymes to translocate PS to the cell surface, also leading to the activity of calpain and gelsolin, which induce actomyosin contraction, allowing plasma membrane budding and the release of the MP PS+ into the extracellular space [36, 37].

Exosomes originate from the intracellular endocytic trafficking pathway, where inward budding of the membrane of late endosomes results in the formation of the ILV through the ESCRT [38, 39]. ESCRT is a protein family that forms complexes (0, I, II, and III) at the MVB membrane to regulate cargo targeting and the formation of the ILV [29, 38]. ESCRT-dependent exosome biogenesis occurs when ESCRT-0 identifies cargo and sorts it into nascent ILVs. Late endosome membrane invagination occurs through the interaction of the ESCRT-0, ESCRT-I, and ESCRT-II complexes. ILV membrane neck contraction is mediated by ESCRT-III and ATPase vacuolar protein sorting-associated protein 4 (VPS4), which favor ILV scission and MVB formation [37, 40–42].

ESCRT-independent exosome biogenesis involves tetraspanins (CD9, the CD63/Syntenin-1 complex, or CD81), which function as signaling molecules to regulate cargo sorting through cholesterol-enriched microdomain formation and interact with transmembrane or cytosolic proteins to induce inward membrane budding. In neuronal cells, ceramide promotes membrane subdomain formation and imposes a spontaneous negative curvature for ILV production. Ceramide generation is mediated by neutral type II sphingomyelinase activity. Additionally, extracellular signals from G protein-coupled receptors, epidermal growth factor receptor (EGFR), tumor necrosis factor receptor (TNFR), the Wnt pathway, or oxidative stress regulate the biogenesis of EVs. ESCRT-dependent and ESCRT-independent pathways synergically act on exosome biogenesis. In this sense, molecules such as ESCRT-associated proteins (Alix and TSG101) and tetraspanins are used as exosome markers to identify their endosomal pathway origin [34–38, 43].

The secretory pathway allows MVBs to fuse with the plasma membrane, favoring the release of ILVs as exosomes. Intracellular trafficking of MVBs is mediated by Rab proteins, whereas the soluble N-ethyl maleimide (NEM)-sensitive factor attachment protein receptor (SNARE) complex drives membrane fusion. Cells can internalize EVs through clathrin-dependent, caveolae-dependent, and receptor-dependent mechanisms, macropinocytosis, phagocytosis, or lipid raft-mediated uptake [38, 44, 45]. Thus, EVs can mediate their intercellular communication functions, inducing phenotypic or behavioral changes in acceptor cells.

### Convergence of EV biogenesis and the RNA virus cycle

Viruses can usurp cellular signaling networks, co-opting exosome biogenesis pathways for their assembly or transferring viral components to establish host permissiveness [46, 47]. In 2003, Gould et al. proposed the Trojan exosome hypothesis, which is based on the retrovirus replication cycle [48], and it has been extended to other RNA viruses [49–55]. The spread of viruses through exosomes is an alternative and effective mechanism for naïve cell infection that modulates the host’s immune response. Convergence of EV biogenesis and the RNA virus replication cycle allows the sorting of viral antigens, genomes, or complete virions as part of the EV cargo. The critical point is membrane fusion during ILV formation: viral NC or complete virion fusion with the ILV membranes or reverse fusion of the ILV with the MVB, releasing virus into the cytoplasm [46, 49].

Focusing on flaviviruses, Hsu et al. first demonstrated in plasma samples from patients with Dengue fever that DENV presented an extra irregularly shaped membrane surrounding a distinct circular vesicle, compatible with EV morphology, with detectable levels of DENV RNA, E, prM, and NS1 proteins. In vitro, these EV-like structures favor the infection of naïve cells, which evade the neutralizing antibody response [56]. These findings suggested that DENV could be carried by EV-like structures such as Trojan horses.

Therefore, do EVs (exosomes and MPs) play a role in the pathogenesis of mosquito-borne flaviviruses? To answer this question, we reviewed the published scientific evidence concerning the functions of EVs in different study models (in vivo, in vitro, and ex vivo) of DENV, ZIKV, YFV, JEV, and WNV infections to establish their role in pathogenesis, especially in severe forms of disease (Table 1). We recognize that evidence from in vitro studies employing cell lines differs from real functions in living organisms; hence, ideal results come from those using primary cells or in vivo or ex vivo models to obtain robust conclusions. However, most of the current data related to EVs and flavivirus diseases are obtained from in vitro studies in cell lines; therefore, elucidating their impact on mosquito-borne flavivirus pathogenesis is important. Further studies using primary cells or in vivo or ex vivo models are necessary.

**Table 1:** Extracellular vesicles in the pathogenesis of dengue virus infection

Viral strain	Model	EV origin cells/fluid	EV type	EV cargo	EV recipient cells	EV function	References
DENV 1–4 from acute DF cases	Ex vivo In vitro	Plasma specimens from DENV-infected patients and human bone marrow cells	EVs CD61+	Viral RNA and proteins (E, prM, NS1)	Hamster kidney fibroblasts (BHK-21) and primary human bone marrow cells	In vitro cell infection and evasion of the neutralizing antibody response	[56]
DENV-2 TVP2176 (El Salvador strain) DENV-2 New Guinea C (NGC) DENV-3 US/BID-V1619/2005	In vitro	Larvae lysate cells (C6/36 HT) from *Ae. albopictus* and (Aag2) from *Ae. aegypti* mosquitos	Exosomes HSP70+	DENV-2 complete RNA genome, C protein mRNA, E/NS1 proteins; DENV-3 C protein mRNA, E protein	C6/36 HT cells, *Cercopithecus aethiops* monkey kidney epithelial (Vero) cells, mouse monocyte-derived dendritic (Mo-DCs) cells, human-skin keratinocytes (HaCaT), and human umbilical vein endothelial (HUVEC) cells	Exosomes from DENV-2- and DENV-3-infected mosquito cells carry viral components. Exosomes from DENV-2-infected mosquito cells mediated viral transmission to mammalian cells through interaction with tetraspanin Tsp29Fb. Inhibition of Tsp29Fb or exosome release are potential therapeutics to block viral transmission	[66]
DENV-2 NGC	In vitro	Larvae lysate cells (C6/36 HT) from *Ae. albopictus* mosquitos	Exosomes CD9-like protein+/CD81-like protein+	Virus-like particles	C6/36 HT cells	Potential trojan vehicles that carry virus-like particles favor naïve cell infection and play a role in viral dissemination	[67]
DENV-2 New Guinea (NG)	In vitro	Larvae lysate cells (ATC-10) from *Aedes aegypti* mosquitos	Exosomes (?)	Mosquito protein cargo (arginase AAEL002675)	–	EVs from DENV-infected mosquito cells have an altered protein cargo with infection-enhancing ability. This protein packaging strategy through the EV pathway promotes viral transmission	[69]
DENV-2 NGC	In vitro	Saliva from *Ae. aegypti* mosquitos	EVs (~ 100–500 nm)	Viral sfRNA	Human hepatoma cells (Huh-7) and neonatal human primary dermal fibroblasts (NHDF)	Salivary EVs from DENV-2-infected mosquitos contain sfRNA. Their stimuli increase viral infectivity and block interferon type I and III signaling	[70]
DENV-2 16,881	Ex vivo In vitro	Platelets from confirmed DENV-infected patients and healthy donors	Microparticles CD41+	Human IL-1β	Human microvascular endothelial cells (HMEC-1)	IL-1β-containing MPs increased vascular permeability (in vitro) and correlated with endothelial dysfunction (ex vivo). Potential biomarkers of severity outcome	[74]
DENV-1 Hawaii DENV-2 16,681 DENV-3 H87 DENV-4 H241	Ex vivo In vitro	Blood specimens from DENV-infected patients (ex vivo); human hepatocellular carcinoma (HepG2) cells (in vitro)	Microparticles PS+, platelet origin (CD41a+), erythrocytic origin (CD235+)	Viral proteins (E, NS1)	–	Ex vivo: (1) Elevated MPs PS+ CD235+ levels correlated with DENV disease severity; (2) Low MPs PS+ CD41a levels are associated with bleeding (DHF). In vitro: DENV infection enhances MPs shedding that could transfer viral components to naïve cells	[75]
DF/SD cases without DENV strain identification	Ex vivo	Blood specimens from DENV-infected patients	Microparticles PS+, platelet origin (CD41a+), erythrocytic origin (CD235+)	–	–	Patients with thrombocytopenia and bleeding manifestations presented decreased levels of MPs PS+ CD41a+. Patients diagnosed as DF with warning signs presented high levels of MPs PS+ CD235a+. The MPs could identify potential progression to severe disease	[76]
DF/SD cases without DENV strain identification	Ex vivo In vitro	Blood specimens form DENV-infected patients	EVs CD41a+ CD63+ CD81+ CD9+ Alix+	Pro- and anti-inflammatory cytokines IL-13, TNF-α, IFN-γ, IL-6, and IL-5	Peripheral blood mononuclear cells (PBMC) and naïve T cells CD4+	Platelet EVs from plasma of severe dengue patients have immunosuppressive properties on T cells CD4+ that may define the progression of dengue disease	[77]
DENV-2 PL046	In vitro In vivo	Primary human and primary mice (PF4-Cre) platelets	EVs CD9+ CD63+ HSP70+ CD41+ CD62p+	–	Primary human and mice neutrophils, primary human monocytes-derived macrophages, human endothelial (HMEC-1) cells C57BL/6 J mice	The EVs from DENV-activated platelets induce neutrophils’ NET formation that contributes to vascular permeability and enhances the release of high concentrations of TNF-α and IL-6 from monocyte-derived macrophages, via activation of CLEC5A/TLR2 receptors on immune cells	[78]
DF/SD cases without DENV strain identification	Ex vivo In vitro	Platelet rich plasma of dengue patients	Exosomes CD41+ CD63+ CD9+	–	Human umbilical vein endothelial cells (HUVEC)	Platelet exosome’s interaction with endothelial cells may promote vascular leakage through proinflammatory mediators release, reducing the vascular barrier integrity	[80]
DF/SD cases without DENV strain identification	Ex vivo	Blood specimens from infected patients	Microparticles HLA-DR+ CD105+	–	–	Association with compromised endothelial stability in DF/SD patients	[82]
DENV-3 5532/290	In vitro	Primary human monocyte-derived dendritic cells (mdDC)	Exosomes CD9+ CD63+ CD81+	Viral E protein and human host RNA (miRNAs, mRNAs)	Mosquito (C6/36) cells and human peripheral blood mononuclear cells (PBMC)	The EVs from DENV-infected mdDC carry viral antigens and favor the infection of naïve mosquito cells. The EVs from IFN-treated PBMC protect naïve cells from viral infection. The RNA profile cargo may be associated with immune dysregulation in SD	[83]
DENV-2 Colombian strain	In vitro	Human adherent (U937) macrophages	Exosomes Alix+ TSG101+ CD63+	Viral NS3 and human miRNA (miR-181a-5p, miR-4301 and miR-4652-3p)	*Macaca mulatta* monkey kidney epithelial (LLC-MK2) cells and human endothelial (EA.hy926) cells	Exosomes did not favor naïve (LLC-MK2) mammalian cells infection. Exosomes transport miRNAs and induce early endothelial barrier changes with a pro-inflammatory (TNF-α, IL-6, and IL-8) activation pattern	[84]
DENV-2 NGC	In vitro	Human monocytes (THP-1) from peripheral blood and human embryonic (HEK293T) cells	EVs TSG101+	Human miR-148a	Human microglial (CHME3) cells	The EVs from DENV-infected cells transfer miR-148a that suppresses the USP33/ATF3 axis in human microglia, promoting neuroinflammation	[89]
DENV-2 UVE/DENV2/2018/RE/47099	In vitro	DENV-2-infected human lung epithelial (A549) cells and NS1-transfected human embryonic kidney (HEK293) cells	Exosomes CD63+ CD81+	NS1 viral protein	–	NS1 dimers secreted into extracellular space are associated with exosomes, reaching privileged sites and thereby acting on pathological processes	[90]
DENV-2 NGC	In vitro	Human umbilical endothelial cells (HUVEC) and human embryonic kidney epithelial (293 T) cells	Exosomes Flotillin-2+ CD63+	Human interferon-inducible transmembrane protein 3 (IFITM3)	Human cervix epithelial (HeLa) cells	The antiviral activity of IFITM3-containing exosomes favors the reduction of DENV-2 entry into host cells	[92]

## EV in mosquito-borne flavivirus pathogenesis

### Dengue virus

#### Dengue fever (DF)

The DENV serotypes are transmitted by *Aedes aegypti* and *Aedes albopictus* mosquitoes [57]. Dengue virus infection can be asymptomatic in most cases [58]. Symptomatic DF can range from a self-limiting acute febrile infection (85–90%) to severe forms (10–15%), known as dengue hemorrhagic fever (DHF) and dengue shock syndrome (DSS) [59, 60]. Currently, DHF/DSS are recognized as severe dengue (SD) characterized mainly by systemic vasculopathy syndrome, capillary leakage, and hemorrhage with loss of vascular integrity [58, 61–65].

Currently, the functions of EVs in DF and SD pathogenesis are unclear. Research findings suggest that they may have roles as intercellular pro- or antiviral signalers that determine the clinical outcome, as prognostic biomarkers, or as potential therapeutic tools.

#### EV from *Aedes* spp. mosquito models

In the mosquito vector‒host interplay, EVs can enhance viral infection. Vora et al. (2018) used an in vitro model of *Aedes albopictus* C6/36 cells infected with DENV-2 (TVP2176 or New Guinea C, NGC) and DENV-3 (US/BID-V1619/2005), isolating EVs HSP70+ that contained complete viral RNA, C, E, and NS1 proteins (DENV-2-infected cells), and E protein and C protein mRNA (DENV-3-infected). Stimuli with EVs from DENV-2-infected cells favor the infection of mosquito and vertebrate (mouse, nonhuman primate, and human) naïve cells. An ortholog (Tsp29Fb) of the tetraspanin CD63 that interacts with the viral E protein was identified, and its blockade reduced mosquito cell infection [66].

Reyes-Ruiz et al. (2019) identified human CD9/CD81 homologs in mosquito cells and demonstrated the presence of DENV-like particles in the ILV structures of DENV-2 NGC-infected C6/36 HT cells. In vitro stimulation with CD9+ CD81+ exosomes from DENV-2 NGC-infected cells favored the infection of naïve cells, indicating their ability to transmit virus [67].

Could EVs be a mechanism of viral transmission through mosquito bites? Cime-Castillo et al. (2015) reported that DENV-2 binds to proteins from *Aedes aegypti* salivary glands and that saliva enhances viral internalization in mammalian cells [68]. In this sense, Gold et al. (2020) hypothesized that EVs from the saliva of DENV-2 New Guinea-infected *Aedes aegypti* have altered protein cargo with infection-enhancing ability in mammalian cells. They isolated EVs flotillin+ HSP70+ from DENV-infected *Aedes aegypti* ATC-10 cells, which contained the arginase AAEL002675. They reported robust enhancement of the viral load in primary dermal fibroblasts previously treated with recombinant arginase [69]. Yeh et al. (2023) were interested in identifying transmission-enhancing factors in mosquito saliva and reported that the EVs in saliva from DENV-2 NGC-infected *Aedes aegypti* Rockefeller colonies contain high levels of sfRNA. The mosquito saliva/EV stimulus resulted in increased virus infectivity in human hepatoma cells and primary dermal fibroblasts [70].

These findings demonstrate that the DENV cycle and exosome biogenesis converge in mosquito cells and that DENV-2-infected cells release EVs that act as Trojan vehicles, transferring viral components, virus-like particles, or mosquito protein cargo that can modify cell permissiveness and favor in vitro infection. More studies are still needed to elucidate the implications of in vivo EVs as efficient mechanisms in mosquito-mediated viral transmission, their impact on vector competence, and their potential as a strategy for mosquito control.

#### Platelet EVs from in vitro and ex vivo models

Platelets, immune cells, and vascular endothelial cells are the main effectors involved in the pathogenesis of DF. Platelet dysfunction can lead to impaired inflammatory responses and tissue damage. Platelet activation, followed by thrombocytopenia, is associated with severe clinical outcomes in viral infections, and activation products that include EVs could be the hallmark during DF/SD [71]. The platelet-derived EVs released during DENV infection may enhance inflammation and endothelial dysfunction. MPs, formerly known as “platelet dust”, have procoagulant activity, and their defects promote bleeding disorders, as described in DHF/DSS [72, 73].

How do platelet-derived MPs contribute to the pathogenesis of DENV infection? Hottz et al. (2013) reported high levels of CD41+ IL-1β+ platelets in patients diagnosed with DF/SD and isolated CD41+ IL-1β+ MPs (from clinical samples and from DENV-2 16,881-stimulated platelets in vitro), with increased levels of the NLRP3 inflammasome and caspase-1 activity. Stimulation with MPs increased the permeability of endothelial cells in vitro. In patients, high hematocrit rates with plasma leakage were correlated with high levels of CD41+ IL-1β+ MPs [74]. These findings suggest that MPs could be used as biomarkers to improve the early detection of severe disease progression.

Punyadee et al. (2015) reported that circulating MPsfrom platelets (CD41a+), erythrocytes (CD235+), monocytes (CD14+), granulocytes (CD66+), T cells (CD3+), B cells (CD19+), and NK cells (CD56+), were present in blood samples of DENV-infected patients with different severities. In acutely ill patients, 50–75% of PS+ MPs are from erythrocytes and platelets and express viral antigens on their surface. Decreased levels of PS+ CD41a+ MPs were associated with a bleeding tendency due to thrombocytopenia. Additionally, they showed in vitro that hepatocellular carcinoma cells infected with DENV-1 Hawaii, DENV-2 16,681, DENV-3 H87, or DENV-4 H241 release PS+ MPs that contain viral E or NS1 proteins on their surfaces [75].

Patil et al. (2018) confirmed that platelet- and erythrocyte-derived MPs are potential biomarkers for predicting the risk of SD progression. In blood samples from patients diagnosed with thrombocytopenia and bleeding manifestations, decreased levels of PS+ CD41a+ MPs were detected, whereas patients diagnosed with DF with warning signs presented increased levels of PS+ CD235+ MPs [76]. In this sense, platelet- and erythrocyte-derived MPs may help identify potential patients who are progressing to SD.

More recently, Kumari et al. (2023) reported in the plasma of patients with mild and severe disease that SD was associated with increased levels of EVs CD41a+ CD63+ CD81+ CD9+ Alix+ from platelets and was highly enriched with the cytokines IL-13, TNF-α, IFN-γ, IL-6, and IL-5. In vitro, EV stimulation strongly suppressed CD4+ T-cell proliferation through the PD-L1/PD-1 interaction. Additionally, EV-stimulated naïve CD4+ T cells expressed high levels of TNF-α and IFN-γ, indicating that EVs promote the Th1 phenotype [77]. Platelet EVs from the plasma of SD patients have immunosuppressive effects on CD4+ T cells, which may define the progression of dengue disease.

Platelets interact with neutrophils, monocytes, and macrophages, activating the innate immune response. Therefore, could platelet-derived EVs promote or modulate immune activation? Sung et al. (2019) reported that DENV-2 PL046-stimulated platelets activate the C-type lectin-like receptor 2 (CLEC-2) signaling pathway, releasing EVs CD9+ CD63+ HSP70+ CD41+ CD62p+. Platelet EV stimuli activate two surface innate immunity receptors (CLEC5A and TLR2) on neutrophils and macrophages, inducing neutrophil extracellular trap (NET) formation with increased vascular permeability in vivo and increasing the secretion of TNF-α and IL-6 from human monocyte-derived macrophages. The authors demonstrated that the simultaneous blockade of CLEC5A/TLR2 reduces NET formation and permeability changes in vivo, suggesting that this intervention is a potential treatment for DF/SD [78, 79]. Focusing on the interplay of platelet-derived EVs with leukocytes, platelet-derived EVs were demonstrated to be efficient host immune effectors and endogenous danger signals that activate CLEC5A and TLR2 receptors on immune cells, which had a cumulative immunomodulatory effect on DENV-mediated CLEC2 activation on platelets, contributing to disease severity. These findings demonstrate that platelet-derived EVs function as signal effectors and not only as vehicles for biomolecules; therefore, blockade of their interaction may also improve the clinical outcome in SD patients [79].

Finally, Vedpathak et al. (2024) isolated platelet-derived CD41+ CD63+ CD9+ CD62p+ exosomes from patients with mild or severe disease. Exosome uptake by endothelial cells (HUVECs) promoted disruption of monolayer integrity with reductions in VE-cadherin and ZO-2 tight junction proteins, and Claudin-1 and PECAM-1 adhesion proteins, indicating the loss of the barrier. Higher levels of C-reactive protein, sVCAM-1, and slCAM-1 were detected both in the cell culture supernatants and in the SD patient samples [80]. The interaction of platelet-derived exosomes and endothelial cells in vitro promotes the release of vascular proinflammatory mediators with the loss of barrier integrity.

These reports highlight the multiple roles of platelet-derived EVs in DF/SD pathogenesis. However, their use as targets for therapies or as biomarkers for severity progression still needs further investigation.

#### Immune cell-derived EVs from in vitro and ex vivo models

Monocytes participate in the establishment of a proinflammatory state, antibody-dependent enhancement (ADE), and vascular endothelium damage, all of which are related to the progression to SD [81]. Naranjo-Gómez et al. (2019) evaluated monocyte subset behavior in DF/SD patient plasma and reported high and low counts of DENV E-positive intermediate and nonclassical monocytes, respectively. The DENV-infected monocytes were activated, expressed intracellular TNF-α and exposed PS at the membrane, which could be associated with MP release. High amounts of HLA-DR+ CD105+ MPs (endothelial origin) associated with compromised endothelial stability were found [82]. The interplay between activated monocytes and MPs from vascular endothelial cells may contribute to the progression to SD.

Do EVs from leukocytes play a role during DENV infection? Martins et al. (2018) reported that DENV-3 5532 (from an SD case) and DENV-3 290 (from a mild DF case) infection of monocyte-derived dendritic cells (mdDC) triggered cell activation and the release of CD9+ CD63+ CD81+ exosomes. Exosomes contain the viral E protein, evade the antibody response, and favor mosquito naïve cell infection. Additionally, exosomes from IFN-treated peripheral blood mononuclear cells (PBMCs) confer resistance to viral infection. The mdDC exosome RNA profile cargo is associated with immune response regulation, the antiviral response, and platelet or endothelial cell activation [83]. Exosomes from DENV-3-infected mdDCs promote pro- or antiviral enhancement, and their RNA profile cargo could be associated with immune dysregulation in SD.

Velandia-Romero et al. (2020) isolated Alix+ TSG101+ CD63+ exosomes from DENV-2-infected macrophages (U937 cells) that transport the viral NS3 protein. They did not find that exosomes favor nonhuman primate cell infection. EV stimulation of endothelial cells (EA. hy926) in vitro modified cell physiology and integrity, upregulated the expression levels of the VE-cadherin and ICAM-1 proteins, and subsequently induced the secretion of high levels of TNF-α, IL-6, and IL-8. Analysis of the miRNA cargo revealed targets associated with cell adhesion, the cell cycle DNA integrity checkpoint, and the regulation of cell communication [84]. There is still no evidence in vivo, through the evaluation of primary cells, that EVs from DENV-2-infected macrophages contribute to endothelial dysfunction. Therefore, further investigations are needed to elucidate this mechanism.

DENV-2 promotes crosstalk between the coagulation and inflammation pathways during DF/SD. Huerta-Zepeda et al. (2008) reported that DENV-2 upregulates tissue factor (TF) in endothelial cells, triggering the generation of hemostatic proteases (thrombin) that activate protease-activated receptors (PARs). Activated PARs initiate inflammatory pathway signaling, which leads to the upregulation of proinflammatory (IL-8) or pro-adherent (VCAM-1) molecules [85]. As described above, the interplay between EVs and endothelial cells promotes early barrier changes, increasing permeability and the loss of barrier integrity; however, the role of EVs in the specific molecular mechanisms of vascular dysfunction is still unclear and poorly understood.

Neurological complications in SD also require consideration; therefore, the WHO endorsed guidelines (2009) to include them in the clinical case classification. Neuropathogenesis is associated with direct viral invasion of the CNS or secondary reactions. Previously, DENV was not considered a neurotropic virus, but clinical reports associating DF with neurological complications have changed this view [86–88].

In this context, could EVs from DENV-infected cells be implicated in the development of DENV neuropathogenesis? Mishra et al. (2020) isolated EVs TSG101+ from DENV-2-infected monocytes (THP-1) and DENV-2 NS1-transfected human embryo kidney (HEK293T) cells, which contained high levels of miR-148a. The stimulation of microglial (CHME3) cells with these EVs reduced the protein expression levels of USP33 and ATF33, both of which are associated with the downregulation of the proinflammatory gene expression pathway. USP33/ATF3 axis dysregulation via EVs miR-148a+ promotes the expression of TNF-α, IFN-β, and NF-κB, which are related to a proinflammatory state. The authors concluded that this model explains why EVs miR-148a+ from DENV-infected cells may contribute to neuroinflammation in vitro [89]. Notably, neuropathogenesis is a complex process, and many factors could be involved. This model of USP33/ATF3 axis dysregulation mediated by EVs is one of many alternative mechanisms that may explain the neurological outcomes described in some SD cases.

#### Other pro- and antiviral functions of EVs in DF/SD

Safadi et al. (2023) reported that DENV-2 strain UVE/DENV2/2018/RE/47099-infected human A549 cells and DENV NS1-transfected HEK293 cells released CD63+ CD81+ exosomes that contained dimers of the viral NS1 protein on their membrane surface. The authors demonstrated that NS1 protein and exosome associations occur in the extracellular compartment and concluded that the association of the NS1 protein with exosomes may increase viral protein stability. Exosomes act as vectors that favor the access of the viral NS1 protein to privileged sites to promote pathological processes, as described for SD [90].

Finally, EVs from infected cells could also contribute to establishing an antiviral state to prevent the spread of infection to neighboring cells [91]. Zhu et al. (2015) evaluated how interferon-inducible transmembrane protein 3 (IFITM3), which is transferred by flotillin-2+ CD63+ exosomes from DENV-2 NGC-infected HUVECs and 293T cells, is expressed in exosome-stimulated HeLa cells, increasing cell resistance to viral infection and significantly reducing DENV-2 entry [92]. IFITM3-containing exosomes serve as vehicles for the cell-to-cell transfer of antiviral resistance, suggesting their potential as a therapeutic strategy.

In summary, EVs from DENV-infected cells play a relevant role in dengue pathogenesis because of their ability to transport different viral contents (RNAs, proteins, or virus-like particles), favoring cell-to-cell transmission and infection. In mosquito‒host interactions, EVs interact with different host cells; EVs can be identified by the presence of human tetraspanin orthologs, and their protein cargo enhances viral binding, allowing host cell infection. In the pathogenesis of DENV infection in humans, EVs from platelets and immunological cells function as Trojan vehicles that favor viral infection, evade the host immune response, and transport active biomolecules (different host/viral RNAs and proteins) that modify naïve cellular behavior. Platelet-derived EVs act as effectors that trigger NET formation in neutrophils and the release of proinflammatory cytokines in monocytes/macrophages via interactions with CLEC5A/TLR2 receptors, promoting vascular endothelial dysfunction in vivo. Additionally, EVs participate in exacerbated inflammatory responses in the CNS or could be implicated in the transfer of antiviral resistance. Knowledge of these mechanisms will lead to further investigations of their potential roles as biomarkers of severity progression (e.g., MPs PS+ CD41a+), therapeutics (e.g., blockade of the interaction of platelet-derived EVs with CLEC5A/TLR2 or use of IFITM3-containing exosomes), or vector control tools.

### Zika virus

#### Zika fever (ZF)

Zika fever (ZF) is a febrile acute disease caused by ZIKV infection and is transmitted by mosquito bites or human-to-human contact. Approximately 80% of infections are asymptomatic and 20% are mildly self-limiting [93–96]. The severe forms of ZF include an expanding spectrum of neurological complications, autoimmune diseases, and congenital Zika syndrome (CZS). ZIKV neurotropism and the susceptibility of immune cells and endothelial barriers allow the virus to reach immunologically privileged sites [97–99]. However, the virus‒cell interaction may not be the only mechanism involved, so the role of the EVs could explain some alternative forms for viral transmission, viral persistence due to the lack of clearance, or immune response avoidance.

#### EV from *Aedes* spp. mosquito models

Could mosquito-derived EVs mediate the cellular changes involved in ZIKV pathogenesis? Martínez-Rojas et al. (2020) isolated PS+ MPs and exosomes CD63-like+ from ZIKV-infected C6/36 cells carrying viral RNA and E protein, promoting the infection of naïve C6/36, Vero, monocyte, and endothelial cells in vitro. EV stimuli promoted intermediate monocyte differentiation and favored endothelial cell damage, establishing a procoagulant, proinflammatory, and proadherent phenotype with the loss of barrier integrity. Stimuli with EVs from ZIKV-infected mosquito cells favored infection, modifying the normal basal state of human cells in vitro [100].

#### EV functions in acute in vitro and in vivo infection models

Focusing on the role of EVs as viral carriers, Safadi et al. (2023) demonstrated that ZIKV PF-25013-18-infected A549 and ZIKV NS1-transfected HEK293 cells released CD63+ CD81+ exosomes containing dimers of the viral NS1 protein on their membrane surface obtained extracellularly in vitro [90]. Similarly, Zhao et al. (2023) reported that ZIKV-infected human umbilical vein endothelial cells released EVs CD63+ CD81+ CD9+ Syntenin+ TSG101+ HSP70+ Alix+ that did not promote infection in vitro despite carrying ZIKV genome RNA, E, C, prM, NS1, and NS5 proteins. The authors demonstrated that E protein-enriched EVs competed with ZIKV virions for binding to neutralizing antibodies, attenuating the ADE effect [101]. Martínez-Rojas et al. (2024) reported that ZIKV MR766-infected monocytes released CD63+ CD81+ TSG101+ Alix+ exosomes. Purified exosomes carried viral antigens (E/NS1 proteins) and viral genomic RNA. Exosomes favored ZIKV transmission and infection, promoting the activation and differentiation of naïve monocytes in vitro and inducing an infection‒activation‒infection cycle that may prolong viral clearance and establish a long-lasting proinflammatory state [102]. These data show that viral elements released as part of the EV cargo may act as an alternative mechanism in virus‒host interactions.

Fikatas et al. (2021) reported that ZIKV PRVABC59-infected human microvascular endothelial cells (hcMECs/D3) released large EVs CD63+ Alix+ TSG101+, which carry the viral NS1 protein. Stimulation with EVs favored cell infection and disturbed the integrity of the cell monolayer. Endothelial EVs are enriched in sphingomyelin and diacylglycerol, which contain more saturated and monounsaturated acyl chains, increasing their membrane rigidity and decreasing their susceptibility to oxidative stress [103]. In vitro, EVs from ZIKV-infected endothelial cells may allow ZIKV to reach anatomic sites such as the CNS to promote neurological disease.

#### EV functions in neurological in vitro and in vivo models

ZIKV infection is associated with CNS damage, which manifests as CZS or Guillain–Barré syndrome. The mechanisms involved in the neuropathogenesis of the Zika virus related to EV functions are poorly understood. Huang et al. (2018) isolated flotillin-2+ Alix+ exosomes from ZIKV (MR766 and PRVABC59)-infected human fetal astrocytes that contained viral RNA. Blockade of the ceramide pathway promoted resistance to ZIKV infection with low exosome release, reducing viral transmission [104]. Zhou et al. (2019) isolated Hsp70+ CD9+ CD63+ exosomes from ZIKV PRVABC59-infected murine fetal primary cortical neurons that contained viral E protein and genomic RNA, which promoted the infection, damage, and death of naïve cells. Additionally, the inhibition of the ceramide pathway reduced infection and exosome release, resulting in exosome-mediated viral transmission in the CNS [105]. Both studies demonstrated that convergence between the viral cycle and exosome biogenesis occurs through the ceramide pathway in CNS models, promoting viral transmission and cell damage.

In this context, York et al. (2021) isolated EVs CD63+ CD9+ HSC70+ populations from ZIKV PRVABC-59-infected SNB-19 cells (i.e., astrocytes) that contained encapsulated viral genome RNA and E protein that promoted infection in naïve cells. The authors demonstrated that CD63 is required in the viral replication cycle; hence, CD63 levels are reduced in infected cells to ensure the release of viral progeny and EVs [106]. This report revealed the role of tetraspanins during EV biogenesis in the modulation of ZIKV infection.

#### EV functions in placental models

In the context of CZS, viral transmission through transplacental infection continues to be a concern. Like other teratogenic viruses, ZIKV must overcome resistance to infection of the placental epithelium constituted by trophoblasts and nontrophoblastic cells, and its innate immunological properties are mediated by the type III interferon (IFN) response [107]. In this context, ZIKV hijacks the antiviral response of trophoblasts to ensure replication cycle completion. Block et al. (2023) demonstrated that ZIKV DAK AR 41524-infected trophoblasts released CD9+ EVs, which contain viral E, NS1, NS4B, and NS5 proteins and diverse host protein cargos that could serve as markers of placenta-specific EVs [108]. Similarly, Lee et al. (2023) investigated how ZIKV infection in trophoblasts manipulates mitochondrial dynamics, promoting the formation of mitochondria-derived vesicles (MDVs). These authors reported that the NS4A protein is responsible for mitochondrial dysfunction, suppressing the IFN response. The ZIKV PRVABC59-infected JEG-3 trophoblasts released CD63+ CD9+ TSG101+ Alix+ MDVs and carried miRNAs involved in the immune response, inflammation, secretion, and autophagy, which may facilitate viral replication in vitro [109]. These findings highlight how ZIKV overcomes placental resistance during trophoblast infection, induces the suppression of the host antiviral response, and favors cell infection.

#### Other pro- and antiviral functions of EVs in the treatment of Zika fever

The immunoregulatory and antiviral effects of EVs could regulate viral pathogenicity through the transfer of specific molecules from infected cells to noninfected neighboring cells. Li et al. (2020) described how CD63+ CD81+ TSG101+ exosomes from ZIKV GZ01-infected human A549 cells contained high levels of host defensin alpha 1B (DEFA1B) mRNA. During ZIKV infection, DEFA1B inhibits viral replication by blocking virus binding, reducing cell proliferation, and slowing cell cycle progression. Exosome-containing DEFA1B stimulation in HEK293T and undifferentiated human neuroblastoma (SH-SY5Y) cells delayed their cell cycles [110]. Therefore, exosomes not only have anti-ZIKV activity but also alter cell cycle progression.

EVs, as transporting vehicles for active biomolecules, have gained relevance as potential therapeutic options because of their ability to cross natural physiological barriers, their intrinsic properties for cell targeting, and their stability in circulation [111]. Zhang et al. (2022) isolated EVs from ZIKV SZ01-infected A6 (type I IFN receptor knockout C57BL/6) mice containing abundant ZIKV-derived small‐interfering RNAs (ZIKV-siRNAs). These EV-specific ZIKV-siRNAs enter the murine bloodstream and are capable of conferring resistance against viral challenge. The authors concluded that these EVs confer RNA‒RNA homology-dependent antiviral activity [112]. Finally, Zhang et al. (2022) designed neuro-specific targeted small CD81+ Syntenin+ EVs containing antiviral siRNAs targeting the NS4A, prM, NS1, and NS3 regions of the ZIKV genome. They reported that these EVs inhibited ZIKV replication. Intravenous administration in pregnant AG6 mice efficiently crossed the placental barrier and reduced the viral loads in mothers and fetuses, protecting both from ZIKV infection and alleviating neuroinflammation and cell damage [113]. These studies demonstrated that targeted delivery via modified EVs is a promising therapeutic alternative that is well tolerated and safe for in vivo models of ZIKV infection.

In summary, in the context of ZF pathogenesis, the roles of EVs include being an efficient mechanism for viral cell-to-cell transmission, a mediator for the establishment of a proinflammatory state, and an effector in neurological or placental cell damage (Table 2). Additionally, EVs mediate antiviral or immunoregulatory activities by transporting specific viral or host biomolecules that confer resistance to infection in naïve cells. This evidence contributes to a better understanding of vector‒virus‒host interactions for new diagnostic or prognostic biomarkers, antiviral therapies, or even vaccine development [114].

**Table 2:** Extracellular vesicles in the pathogenesis of Zika virus infection

Viral strain	Model	EV origin cells/fluid	EV type	EV cargo	EV recipient cells	EV function	References
ZIKV MR766	In vitro	Larvae lysate cells (C6/36) from *Aedes albopictus* mosquitos	Microparticles PS+ and exosomes CD63-like+	Viral RNA Viral E protein	Naïve C6/36 cells, monkey kidney (Vero) cells, human monocytes (THP-1), human endothelial (HMEC-1) cells	The EVs from ZIKV-infected C6/36 cells favor naïve mosquito and mammalian cell infection. The EV stimuli induce monocyte activation and differentiation, and the endothelial cell damage with increased barrier permeability	[100]
ZIKV PF-25013–18	In vitro	ZIKV-infected human lung epithelial (A549) and NS1-transfected human embryo kidney (HEK293) cells	Exosomes CD63+ CD81+	NS1 viral protein	–	The NS1 dimers secreted into extracellular space are associated with exosomes	[90]
ZIKV without strain identification	In vitro In vivo	Human umbilical vein endothelial cells (HUVEC)	Exosomes CD63+ CD81+ CD9+ Syntenin+ TSG101+ HSP70+ Alix+	Viral genome RNA and proteins E (highly enrichment), C, prM, NS1, and NS5	Naïve Vero E6 cells, human lymphoblasts (K562), and A6 mice (Ifnar1^−^/^−^ C57BL/6, 6 weeks old)	Exosomes from ZIKV-infected endothelial cells carry viral elements but do not favor viral transmission/infection. The high E-enrichment allows high affinity binding to neutralizing antibodies, attenuating the ADE	[101]
ZIKV MR766	In vitro	Human monocytes (THP-1)	Exosomes CD63+ CD81+ TSG101+ Alix+	Viral E and NS1 proteins, genomic RNA transcripts	Naïve THP-1 cells and monkey kidney (Vero cells)	Purified exosomes from ZIKV-infected monocytes favor viral transmission, infection, and the differentiation/activation of naïve cells, promoting an infection-activation-infection cycle	[102]
ZIKV PRVABC59	In vitro	Human brain microvascular endothelial cells (hcMEC/D3)	Large EVs CD63+ Alix+ TSG101+	Viral NS1 protein	Naïve hcMEC/D3	The EVs from ZIKV-infected endothelial cells can be used as viral element carriers, enhancing ZIKV transmission and infection to susceptible cells and altering the monolayer integrity	[103]
ZIKV MR766/ PRVABC59	In vitro	Primary human fetal astrocytes	Exosomes Flotillin2+ Alix+	Viral RNA	–	Inhibition of neutral sphingomyelinase-2 activity induces resistance to infection and reduces the exosome biogenesis	[104]
ZIKV PRVABC59	In vitro	Primary murine fetal cortical neurons	Exosomes HSP70+ CD9+ CD63+	Viral RNA Viral E protein	Naïve murine fetal cortical neurons	Naïve cells infection, damage, and death. ZIKV modulates neutral sphingomyelinase-2 SMPD3 activity in cortical neurons for its infection: inhibition of SMDP3 activity (by GW4869) or silencing of *smdp3* gene (by siRNA) reduces viral burden and transmission through exosomes	[105]
ZIKV PRVABC-59	In vitro	SNB-19 (astrocytes) cells	Small EVs CD63+ CD9+ HSC70+	Encapsuled viral genomic RNA and E protein	Naïve SNB-19 cells	The EVs from ZIKV-infected astrocytes favor the infection of naïve cells. Infected cells present a dysregulated tetraspanin pattern: CD63 is necessary for EV and ZIKV release	[106]
ZIKV DAK AR 41524	In vitro	Rhesus macaque trophoblast stem cells	EVs CD9+	Viral E, NS1, NS4B, and NS5 proteins with diverse cell mRNA, miRNA, and protein cargos	–	The EV cargo from ZIKV-infected trophoblasts could be used to noninvasively identify placental ZIKV infection in the first trimester of pregnancy	[108]
ZIKV PRVABC59	In vitro	JEG-3 trophoblast cells	Small EVs (MDVs) CD63+ CD9+ TSG101+ Alix+	Host mitochondrial and miRNAs involved in immune response, inflammation, secretion, and autophagy	–	The small MDVs release is a hijacked mechanism during ZIKV infection, inducing the suppression of the antiviral response and favoring cell infection	[109]
ZIKV GZ01	In vitro	Human adenocarcinoma alveolar basal epithelial cells (A549)	Exosomes CD63+/CD81+/TSG101+	Human host mRNA (DEFA1B)	Human embryonic kidney epithelial cells (HEK293T), undifferentiated human neuroblastoma cells (SH-SY5Y)	The ZIKV infection inhibition through blocking viral attachment to target cells and retarding the progression of cell cycles	[110]
ZIKV SZ01	In vivo	Serum from A6 (type I IFN receptor knockout C57BL/6)	EVs not characterized	ZIKV-derived small‐interfering RNAs (ZIKV-siRNA)	Naïve A6 mice	The ZIKV-siRNA confer resistance against viral challenge	[112]
Not used for EV production	In vitro In vivo	Human embryonic kidney (HEK) 293 T cells	Small EVs CD81+ Syntenin+	Designed siRNA targeting four distinct regions (NS4A, prM, NS1, NS3) of the ZIKV genome	ZIKV SZ01-infected A549 and Vero cells; AG6 mice (8 weeks old) or pregnant mice (E17.5)	Modified small EVs encapsulating siRNA were able to suppress the ZIKV replication in vitro. Also, these EV efficiently penetrated the placental barrier, reducing ZIKV load and protecting both the mother and the brain of fetuses	[113]

### Yellow fever virus

#### Yellow fever (YF)

Currently, YFV infection can be controlled by a highly effective virus-attenuated vaccine. Therefore, yellow fever cases occur mainly in unvaccinated populations [115]. Actual evidence indicates that the role of EVs in YF pathogenesis is hypothetical.

#### EV functions from in vitro models

Sinigaglia et al. (2018) reported that immature virions and capsid-free RNA from YFV 17D-204-infected hepatocytes activated plasmacytoid dendritic cells (pDCs) to produce IFNs in a TLR7- and cell contact-dependent manner. They proposed that YFV RNA cell-to-cell transfer occurs through carriers such as PS-enriched vesicles (compatible with EVs) that evade the host immune response. The authors demonstrated the presence of membranous viral clusters at sites of contact between YFV-infected cells and pDCs [116]. The main limitation of this report was the lack of identification of EV membrane markers; however, these findings suggest that membranous vesicles such as EVs may be involved in cell activation and viral cell-to-cell transmission.

Previously, in 2011, Carpp et al. demonstrated that the viral NS3 protein interacted with the host Alix protein to contribute to the release of infectious viral particles. In an in vitro model of YFV (17DD)-infected Vero cells, NS3 and Alix colocalized in the perinuclear region, forming a protein complex required for virion release. They also proved that the truncated forms of Alix inhibited the release of YFV without affecting its replication [117]. Despite not focusing on EVs, the molecular findings are relevant because cells respond to cross-signaling between viral and host proteins, which are essential for virion and EV release.

In summary (Table 3), the potential role of EVs in YFV pathogenesis is hypothetical. As demonstrated for DENV and ZIKV, EVs may act as viral carriers or be necessary for viral release through the interaction between viral proteins and the host ESCRT-associated proteins that execute cargo sorting during exosome biogenesis. These findings demonstrate that specific steps during EV biogenesis are necessary to complete the YFV cycle and facilitate its spread to target cells, in which processes EVs can be important mediators.

**Table 3:** Extracellular vesicles in the pathogenesis of yellow fever virus infection

Viral strain	Model	EV origin cells/fluid	EV type	EV cargo	EV recipient cells	EV function	References
YFV 17D-204	In vitro	Hepatocytes (Huh7.5 cells)	EVs without identification	Viral RNA or viral immature particles	Plasmacytoid dendritic cells	Hypothetical: Cell-to-cell viral carriers	[116]
YFV 17DD	In vitro	Vero cells	–	–	–	Hypothetical: YFV NS3 interacts with host Alix protein to contribute for viral release. Alix executes cargo sorting in exosome biogenesis	[117]

### Japanese encephalitis virus

#### Japanese encephalitis (JE)

JE is responsible for a greater burden of neurological disability than any other arthropod-borne disease. Infection can be prevented by vaccination. Currently, there are no specific antivirals, and only supportive treatment is available [118–120]. Recent studies have demonstrated that EVs are involved in viral infection-mediated neuroinvasive diseases [53, 121]. However, the functions of EVs in JE pathogenesis remain elusive.

#### EV functions from in vitro and in vivo models

In 1987, Hase et al. described the maturation process of the JEV SA-14 strain in mosquito C6/36 cells and murine brain cells. They reported that virions were enclosed in vesicles distributed throughout the cytoplasm and transported to the cell surface to be released extracellularly [122]. Interestingly, the authors confirmed Leary and Blair’s postulate that JEV uses secretory-type exocytosis [123], the same pathway that was later described for exosome release. This report revealed that during the viral cycle, vesicle formation is necessary to ensure virion maturation and release. Later, convergence with EV biogenesis will be demonstrated.

Flavivirus E and NS1 proteins are cotransformally processed during virion maturation through the cell secretory pathway [124]. Mason (1989) demonstrated that in Vero cells, the E and NS1 proteins are released at a slow rate, whereas in C6/36 cells, the NS1 protein is not released. A key finding related to EVs is the amphipathic nature and sedimentation properties of the NS1 protein found in mammalian cell culture supernatant, suggesting that NS1 contains membrane-binding domains and is released from infected cells in a membrane-associated particulate form, as described in other flaviviruses. The authors concluded that extracellular membranous particles containing the NS1 protein could be related to the strong immunological response observed in JE [125]. This report constitutes the first approach for the potential role of EVs as viral element carriers implicated in JE pathogenesis.

In summary (Table 4), in the context of JEV pathogenesis, EVs have been proposed to be necessary for viral maturation and extracellular virion release; additionally, as viral components (NS1 protein) carriers, EVs can induce immune responses, as observed in JE cases.

**Table 4:** Extracellular vesicles in the pathogenesis of Japanese encephalitis virus infection

Viral strain	Model	EV origin cells/fluid	EV type	EV cargo	EV recipient cells	EV function	References
JEV S-14	In vitro In vivo	C6/36 cells and ICR mice	Intracellular vesicles	Viral particles	–	Hypothetical: EV biogenesis is associated with viral maturation. Viral particles appeared to be carried within vesicles to the cell surface to be released extracellularly	[122]
JEV Nakayama	In vitro	Vero cells and C6/36 cells	Intracellular vesicles	Viral proteins (E, NS1)	–	Hypothetical: JEV NS1 protein contains membrane-binding domains, and it is released from infected cells in a membrane-associated particulate form	[125]

### West Nile virus

#### West Nile fever (WNF)

WNF may progress to a neuroinvasive disease in elderly immunocompromised patients. To date, no WNV-specific antiviral treatments or vaccines are available. Like other mosquito-borne flaviviruses, prevention depends on organized and sustained mosquito control with public education [126, 127]. In this context, what is the role of EVs in WNV neuropathogenesis?

#### EV functions from in vitro models

The role of EVs as a mechanism for WNV genomic RNA transmission was proposed by Zhou et al. (2018). They used an in vitro model of WNV CT2741-infected Neuro-2a cells and isolated CD9+ HSP70+ exosomes that contained the viral E gene transcript, suggesting that WNV genomic RNA is a part of their cargo. The authors concluded that the exosomes from neuronal cells mediate the cell-to-cell transmission of WNV RNA, which may favor infection in a receptor-independent manner in the CNS [128].

As described for other flaviviruses, the cell-to-cell signaling mediated by EVs in the host innate immune response should be considered a mechanism of EV antiviral or proinflammatory activity. Slonchak et al. (2019) used an in vitro model with WNV Kunjin-infected human A549 cells to isolate CD9+ CD63+ HSP70+ exosomes containing specific host RNAs (miRNAs, small noncoding RNAs (sncRNAs), and mRNAs) associated with virus‒host interactions, inflammation, and the innate immune response [129]. This report revealed that WNV infection altered the RNA profile of the EV cargo (Table 5).

**Table 5:** Extracellular vesicles in the pathogenesis of West Nile virus infection

Viral strain	Model	EV origin cells/fluid	EV type	EV cargo	EV recipient cells	EV function	References
WNV CT2741	In vitro	Mouse Neuro-2a cells	Exosomes CD9+ HSP70+	Viral RNA	Mouse Neuro-2a cells	Viral RNA cell-to-cell transmission	[128]
WNV Kunjin	In vitro	Human A549 cells	Exosomes CD9+ CD63+ HSP70+	Human host RNA profile (miRNAs, sncRNAs, and mRNAs)	Human A549 cells transfected with small RNAs from EV isolated of WNV-infected cells	WNV infection stimulates the incorporation of increased levels of specific host RNA profile into EVs that regulates genes associated with viral and inflammatory processes. Small RNAs from EVs induced innate immune response on naïve cells	[129]

In summary, during WNV infection, EV functions are associated with the transport of viral RNA, which is implicated in cell-to-cell transmission. Additionally, WNV-infected cells release EVs containing host RNAs associated with antiviral and proinflammatory functions. The interplay between EVs and naïve cells may promote viral transmission and/or immunostimulatory activity.

## Conclusions and perspectives

For the control of mosquito-borne flaviviruses, it is necessary to understand how viruses interact with different host cells. Knowledge of the molecular or cellular pathways involved in vector‒virus‒host interactions that allow viruses to replicate, persist due to immune evasion, or establish a prolonged inflammatory state is critical for determining disease outcomes.

This review aimed to highlight the currently available data only without the claim of representing established knowledge. The current data related to EVs and flavivirus diseases are mainly obtained from in vitro studies in cell lines; thus, elucidating their impact on mosquito-borne flavivirus pathogenesis is important. Further studies using primary cells or in vivo or ex vivo models are necessary.

Based on the ability of EVs to transfer viral components or active biomolecules, the EVs released from infected cells represent a novel and efficient mechanism for viral (DENV, ZIKV, YFV, JEV, and WNV) cell-to-cell transmission through their ability to carry and transfer different viral contents, such as RNAs, proteins, or viral particles, as well as the ability to evade the host immune response. Convergence between EV biogenesis and the flavivirus replication cycle is critical because viral and host molecules interact; thus, this process has potential as a target for antiviral therapy. From a vector perspective, EVs mediate efficient transmission and favor virus adaptation in vertebrate hosts, ensuring the maintenance of the viral cycle. From the host perspective, as a response to infection, EVs may have altered RNAs and protein cargoes that modify naïve cell behavior, enhancing the inflammatory response and tissue damage or mediating a protective effect through the transfer of antiviral resistance. Additionally, EVs participate in exacerbated inflammatory responses in the CNS or placenta or could be implicated in the transfer of antiviral resistance.

EVs can also stimulate cell signaling pathways, as described for platelet-derived EVs, which trigger NET formation and the release of proinflammatory cytokines in immune cells, promoting vascular dysfunction in vivo. In this sense, EVs not only function as transport vehicles but also interact with cell receptors to promote changes in cell behavior.

Knowledge of these mechanisms will lead to further investigations to develop novel strategies employing EVs to combat viral infection and reduce disease severity based on their potential roles as biomarkers of severity progression, therapeutics (e.g., blockade of EV biogenesis or their interaction with cell receptors), or vector control tools.

Therefore, currently available data concerning EVs evaluated in different models suggest that they may contribute directly to the pathogenesis of mosquito-borne flaviviruses.

Future research will contribute to understanding the role of EVs, especially their mechanistic and functional effects in vivo, to elucidate their real impact on mosquito-borne Flavivirus diseases. The above could be a near reality owing to the rapid evolution of the field of extracellular vesicles.

## Data Availability

Not applicable.

## Abbreviations

ADE: Antibody-dependent enhancement
AES: Acute encephalitis syndrome
Arbovirus: Arthropod-borne virus
BM: Bone marrow
CD: Cluster of differentiation
CDC: Centers for Disease Control and Prevention
CFS: Cerebrospinal fluid
CNS: Central nervous system
CZS: Congenital Zika syndrome
DCs: Dendritic cells
DENV: Dengue virus
DF: Dengue fever
DHF: Dengue hemorrhagic fever
DSS: Dengue shock syndrome
ER: Endoplasmic reticulum
ESCRT: Endosomal sorting complex required for transport
EVs: Extracellular vesicles
HUVEC: Human umbilical vein endothelial cells
ICAM-1: Intercellular adhesion molecule 1
IFITM3: Interferon-inducible transmembrane protein 3
ILV: Intraluminal vesicles
ISEV: International Society for Extracellular Vesicles
ISG: Interferon stimulating genes
JEV: Japanese encephalitis virus
LGTV: Langat virus
mdDC: Monocyte-derived dendritic cells
MDV: Mitochondrial derived vesicles
miRNA: Micro ribonucleic acid
MISEV: Minimal information for studies of extracellular vesicles
MPs: Microparticles
mRNA: Messenger ribonucleic acid
MVB: Multivesicular bodies
NC: Nucleocapsid
NLRP3: Nucleotide-binding domain leucine-rich repeat-containing protein type 3
NS: Non-structural
ORF: Open reading frame
PAHO: Pan American Health Organization
PBMC: Peripheral blood mononuclear cells
PE: Phosphatidylethanolamine
PECAM-1: Platelet endothelial cell adhesion molecule
PS: Phosphatidylserine
RBP: RNA-binding proteins
RNA: Ribonucleic acid
SD: Severe dengue
sfRNA: Sub-genomic flaviviral RNA
sICAM-1: Soluble intercellular adhesion molecule 1
siRNA: Small interfering ribonucleic acid
sncRNA: Small non-coding ribonucleic acid
sVCAM-1: Soluble vascular cell adhesion molecule 1
TEM: Transmission electron microscopy
TF: Tissue factor
TLR2: Pattern recognition receptor toll-like receptor 2
TNF-α: Tumor necrosis factor alpha
UTR: Untranslated region
VCAM-1: Vascular cell adhesion molecule 1
WHO: World Health Organization
WNV: West Nile virus
YFV: Yellow fever virus
ZF: Zika fever
ZIKV: Zika virus
